# Endocrine-Disrupting Effects of Bisphenol A on the Cardiovascular System: A Review

**DOI:** 10.3390/jox12030015

**Published:** 2022-07-13

**Authors:** Maria Inês Fonseca, Margarida Lorigo, Elisa Cairrao

**Affiliations:** 1 CICS-UBI, Health Sciences Research Centre, University of Beira Interior, 6200-506 Covilhã, Portugal; mariainesf246@gmail.com (M.I.F.); margarida.lorigo@gmail.com (M.L.); 2 FCS-UBI, Faculty of Health Sciences, University of Beira Interior, 6200-506 Covilhã, Portugal

**Keywords:** BPA, plasticizer, endocrine disruptor, cardiotoxicity, human health

## Abstract

Currently, the plastic monomer and plasticizer bisphenol A (BPA) is one of the most widely used chemicals. BPA is present in polycarbonate plastics and epoxy resins, commonly used in food storage and industrial or medical products. However, the use of this synthetic compound is a growing concern, as BPA is an endocrine-disrupting compound and can bind mainly to estrogen receptors, interfering with different functions at the cardiovascular level. Several studies have investigated the disruptive effects of BPA; however, its cardiotoxicity remains unclear. Therefore, this review’s purpose is to address the most recent studies on the implications of BPA on the cardiovascular system. Our findings suggest that BPA impairs cardiac excitability through intracellular mechanisms, involving the inhibition of the main ion channels, changes in Ca^2+^ handling, the induction of oxidative stress, and epigenetic modifications. Our data support that BPA exposure increases the risk of developing cardiovascular diseases (CVDs) including atherosclerosis and its risk factors such as hypertension and diabetes. Furthermore, BPA exposure is also particularly harmful in pregnancy, promoting the development of hypertensive disorders during pregnancy. In summary, BPA exposure compromises human health, promoting the development and progression of CVDs and risk factors. Further studies are needed to clarify the human health effects of BPA-induced cardiotoxicity.

## 1. Introduction

According to the World Health Organization, cardiovascular diseases (CVDs) are the leading cause of death worldwide. The majority of CVDs are chronic and asymptomatic over a long time, and usually, the first symptoms only appear as the disease progresses. However, CVDs can also induce immediate sudden death, which is the main cause of premature mortality worldwide. It is estimated that by the year 2030, 23.6 million people will die from CVDs each year. However, there is a slight downward trend in mortality and CVD incidence in north-eastern and Southern Europe [1].

Currently, the influence of environmental contaminants on humans has been proposed as a cause of CVDs [2]. Every year, millions of tons of plastic are produced worldwide which results in continuous daily human exposure to these environmentally toxic chemicals [3]. Endocrine-disrupting compounds (EDCs) are defined by the North American Environmental Protection Agency as a natural or synthetic compounds which can interfere with the actions of the endocrine system. Specifically, EDCs can mimic or antagonize the action of endogenous hormones and alter their synthesis, transport, binding, and elimination. Then, these emerging compounds can disrupt normal hormonal homeostasis, reproduction, and/or behavior [4,5]. Moreover, EDCs are potential modulators of cardiovascular physiology, from which emerges the need for the study of their cardiotoxicity [6].

**Figure 1:**
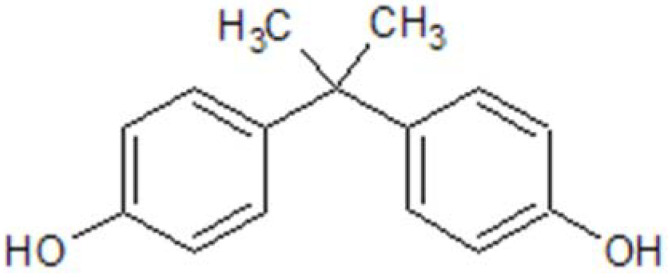
BPA chemical structure, drawn in ChemDraw^®®^.

Among the various EDCs, bisphenol A (BPA) (Figure 1) stands out as one of the most widely produced EDCs worldwide [7]. BPA, also designated as 4,4’-ispropylidenediphenol by IUPAC, is a synthetic organic compound formed of two phenol groups, used in polycarbonate plastics and epoxy resins [8]. In 1891, BPA was first synthesized by the chemist Alexender P.Dianin, and about 40 years later, some of its estrogenic effects began to be discovered [9]. Its properties give plastics greater thermal resistance and elasticity, and for this reason, BPA is still in use after 130 years of its discovery.

Regarding its appearance, BPA is a solid, white, crystalline substance whose melting point is 156 °C, with a boiling point of 220 °C (at a pressure of 5 hPa). Furthermore, BPA has a water–octanol coefficient of log Pow = 3.32, indicating that it has good solubility in fats, and contrariwise, low solubility in water (~200 mg/dL^3^ at 25 °C). The presence of hydroxyl groups determines the good reactivity of BPA. Like other phenols, bisphenol can be converted into ethers, esters, and salts [10]. The structure of BPA is similar to that of 17β-estradiol, and for that reason, this EDC binds to estrogenic receptors such as ERα, ERβ, ERγ, G-protein-coupled estrogen receptor (GPR30), and peroxisome proliferator-activated receptor gamma (PPAR-γ) [11]. Although the mechanisms of action are not yet fully understood, BPA has been shown to induce insulin resistance, adipogenesis, pancreatic β-cell dysfunction, inflammation, and oxidative stress [12].

Therefore, given its ubiquity and endocrine-disrupting (estrogenic) properties, daily exposure to BPA has become a major public health concern, and it is even considered that the cardiovascular system is highly susceptible to the disruptive effects of BPA [13]. Indeed, several studies have associated BPA exposure with an increased risk of developing CVDs via different intracellular mechanisms (as will be described in this review). Thus, our aim is to address the disrupting effects of BPA in the human cardiovascular system, reviewing the current literature based on epidemiological data and experimental studies in humans and animals, with a focus on the underlying molecular mechanisms.

## 2. Approach to the Review

Recent studies regarding the cardiovascular effects of BPA on animal and human models will be presented in this review. A literature review was carried out for epidemiological and experimental data on the cardiovascular system and supported by in vitro studies. A PubMed search on articles published between the years 2011 and 2022 was carried out. The database search was performed using a combination of terms relating to bisphenol A (“bisphenol A”, ”BPA”, “endocrine disruptor compound”, and “plastic contaminants”), to the cardiovascular system (“cardiovascular system”, “arteries”, “vascular”, “smooth muscle”, “vascular smooth muscle”, “smooth muscle cells”, “endothelium”, and “heart”), and to cardiovascular outcomes (“cardiovascular diseases”, “hypertension”, “endothelial dysfunction”, ”atherosclerosis”, ”myocardial infarction”, “heart failure”, “heart rate variability”, “blood pressure”, and “peripheral vascular disease”). In addition to these terms, we also included in the search relevant citations of the articles used. From all the articles retrieved, duplicates, unrelated, and inaccessible papers were excluded. This review was performed following a weight-of-evidence approach, and the results of the most important studies and those with greater relevance for this paper are described below.

## 3. Exposure to BPA

Globally, the use of BPA has progressively increased, reaching more than 10 million tonnes per year [7,10]. BPA is present in 95% of products requiring epoxy resins and polycarbonates, such as food containers, bottles, toys, dental products, CDs, DVDs, and water pipes [14]. The use of BPA in consumables and medical products makes its exposure continuous, having been detected, for example, in urine in over 90% of the United States (US) population [15]. In addition, BPA has also been identified in other biological samples, such as maternal blood (0.3 to 18.9 ng/mL) [16,17,18,19], maternal urine (31.9 μg/L) [19], amniotic liquid (median = 0.26 ng/mL) [17], placental tissue (median = 12.7 ng/g) [16], umbilical cord blood (0.2 to 9.2 ng/mL) [16,18,20], breast milk (0.61 to 0.7 μg/L) [19,21], and human colostrum (3.41 ng/mL) [22]. However, in biomonitoring studies, urinary samples of BPA are often used. The reason is that BPA is a non-persistent chemical, so its chemical concentration is higher in these samples, compared to human plasma or serum [6,23]. Nevertheless, the degree of exposure to BPA is quite variable depending on socioeconomic factors, lifestyle, medical status, and exposure pathways [6]. With regard to this, oral exposure is considered the most prevalent, with BPA levels associated with dietary choices [6,24]. On the other hand, cutaneous absorption and/or inhalation may also be associated with a higher level of exposure to unconjugated or biologically active BPA, which may persist for longer periods (~5.4 h) compared to ingested, subject to first-pass metabolism [23].

BPA, similarly to other EDCs, interacts with receptors activated by estrogens, androgens, thyroid hormones, and peroxisome proliferator, and acts as an agonist or antagonist via a receptor-dependent signaling pathway; this is attributed to its chemical structure. However, its chemical structure may be an advantage, as demonstrated for binding to ER, in which BPA does not achieve proper accommodation in the confines of the hormone-binding site (it only induces a displacement of α-helices forming the ligand-binding domain (LBD)) [11]. Moreover, Tan et al. demonstrated that EDCs share three levels of key fragments: primary and secondary fragments (responsible for the receptors binding, which discriminate active and inactive compounds), and tertiary fragments that determine their activity type (agonist, antagonist, or agonist–antagonist (A-Anta)). This determination is achieved via the interaction of EDCs with the functional lobes, directly affecting the AF-2 surface, which is responsible for coregulator recruitment. In the case of BPA, this EDC contained primary fragments of oxygen-containing aromatics and secondary ones (bisphenol group) [25]. The coexistence of primary and secondary fragments is responsible for activating BPA (active compound). Activation of the estrogen receptor (ER) and androgen receptor (AR) is achieved via interactions of the secondary fragment of stabilized BPA conformations in the LBD by forming hydrogen bonds with R394 amino acid and via van der Waals interactions with N705 amino acid, respectively. The comprehension of secondary fragments forming interacting networks with LBD amino acids is the basis for the activity of BPA. Ligand fragments of BPA interact with LBD and cause changes in the conformation of the AF-2 surface, recruiting two cofactors and, thus, determining its tertiary fragment (A-Anta activity).

Similar to natural hormones, some of the experimental studies with BPA suggest a non-monotonic response, highlighting that risk assessment is required with exposures from ‘lower’ to ‘higher’ doses, given the characteristic U-shaped response also observed by other EDCs [26]. Not surprisingly, this property can complicate BPA toxicity risk assessment, as this EDC can interact with hormone receptors in specific cell types and/or have multiple biological endpoints with linear dose–response that collectively produce a non-monotonic dose–response relationship [27].

Over the past few years, there has been growing concern regarding the adverse effects of BPA exposure on human health. These adverse effects have led countries such as Denmark and Belgium to restrict the use of BPA in food packaging for children between the ages of 0 and 3. Sweden has also limited the use of BPA in varnishes and food packaging coatings for children in the same age group. In addition, Austria has restricted the use of BPA in pacifiers and bottles since October 2010 [28].

Therefore, given the characteristics of BPA as an EDC and its continued exposure to humans, in the following sections, the endocrine-disrupting effects of BPA on the cardiovascular system will be described for animal models (Section 4) and human models (Section 5) (please see below).

## 4. Effects of BPA on Animal Models

Many studies have linked BPA exposure to adverse effects on health, mainly in reproductive organs; neural, immune, and metabolic systems; and cancer [29,30]. Nevertheless, recent evidence further revealed the relationship between BPA exposure and the incidence of cardiovascular disease, myocardial infarction, hypertension, and altered cardiac electrophysiology [27,31].

Recently, Lind et al. established several key characteristics of cardiovascular toxicants in research: (1) drug discovery, (2) environmental health hazard assessment, (3) research biomarkers (for epidemiological studies and clinical trials), and (4) clinical practice. Thus, the author defined several techniques using in vitro, in vivo, and ex vivo models that are currently used to classify a substance as cardiotoxic. In this review, we will try to address the effects of BPA in these various aspects [32].

### 4.1. In Vitro Studies

BPA exposure has been extensively studied in rodents, fish, and canine animals. In vitro studies can offer a quicker and more flexible approach to health effects and are also an indispensable tool for studying mechanistic pathways.

As previously mentioned, ion channels play key roles in the excitability of cardiac cells (the sinoatrial node, atria, atrioventricular node, Purkinje fibers, and ventricles cells), and in the regulation of vascular smooth muscle and in endothelial cells. Thus, any alteration in the activity or structures of ion channels in these cells may induce cardiovascular pathologies [33,34].

Regarding the effect of BPA on ion channel activity, Asano et al. was the first to perform a study on human and canine coronary SMC, and showed that BPA (10 µmol/L) activates large conductance Maxi-K channels (BK_Ca_) in a non-genomic pathway. BK_Ca_ activation depend on the two main subunits of this channel (α-subunit and β1 subunit). The α-subunit alone was sufficient for the response of these channels to BPA; however, in the presence of the regulatory subunit (β1 subunit), the response to BPA was improved. Thus, the authors concluded that the rapid effect of BPA was similar to that observed for estradiol, and suggested that the effect of BPA could provoke a vasodilatory effect on coronary arteries through the opening of Maxi-K channels [35]. A few years later, in 2014, Rottgen et al. demonstrated, in a genomic study, that BPA (100 µmol/L) activates the BK channels through an increase in α- and β1-subunits expression, corroborating the work of Asano et al. Indeed, it was concluded that BPA activates BK channels via an extracellular binding site and via an intracellular binding site that depends on the presence of the β1 subunit [36]. Both studies, Rottgen et al. and Asano et al., suggested that BPA induces a vasorelaxant effect. More recently, in 2017, in rat aorta, the BPA vasorelaxant effect was proven, and the authors also showed, via patch clamp in A7r5 cells—a vascular smooth muscle cell line obtained from embryonic rat aorta—that BPA induces an inhibition in the voltage-dependent calcium (Ca^2+^) influx currents via a non-genomic pathway. Moreover, the authors also demonstrated that these Ca^2+^ currents were due to the L-type Ca^2+^ channels [37].

Concerning the BPA effect in the ionic channels from cardiomyocytes, Deutschmann et al. showed—in rat GH3 cells, mouse dorsal root ganglion neurons or cardiac myocytes, and recombinant human R-type Ca^2+^ channels expressed in human embryonic kidney (HEK) 293 cells—that BPA (1–100 µmol/L) can rapidly and reversibly inhibit Ca^2+^ currents through native L-, N-, P/Q-, T-type Ca^2+^ channels [38]. The following year, Michaela et al. corroborated this effect of BPA (1–100 μmol/L) on T-type Ca^2+^ channels, these channels being similar to those expressed in nodal and conduction cardiac cells [39]. The inhibition of L-type Ca^2+^ channels was also shown by Liang et al. [40] in ventricular cells, and by Hyun et al. in human-induced pluripotent stem-cell-derived cardiomyocytes (hiPSC-CMs). Moreover, this author also observed that BPA (1–100 µmol/L) inhibited Nav1.5 and hERG channel activity [41]. More recently, the effect of BPA was compared with BPA substitutes, BPF and BPS; the inhibitory effect on the voltage-gated sodium channel (Nav1.5), L-type voltage-gated Ca^2+^ channel (Cav1.2), and the rapidly activating delayed rectifier potassium channel (hERG) was greater for BPA [42]. Moreover, we can mention that the effect of BPA on sodium (Na^+^) and potassium (K^+^) channels has been analyzed previously by other authors [41,42,43,44].

The regulation of the cardiovascular system, as mentioned above, does not depend exclusively on the modulation (inhibition or activation) of the ion channels. The phosphorylation of key regulatory proteins is also preponderant in cell signaling pathways that control the concentration of intracellular and extracellular ions, of which Ca^2+^ is the most important. Thus, it has also been shown by some studies that BPA can act to modify these pathways [27].

The exposure of rat cardiomyocytes to BPA and 17β-oestradiol (E2) shows that these compounds rapidly promoted arrhythmogenesis in cardiac myocytes, and those actions were mediated by the alteration of myocyte Ca^2+^ cycling. The effects of each compound on contractility were female-specific, that is, the contractility of male cardiomyocytes was not affected by either BPA or E2 [45,46,47]. Thus, Yan et al. demonstrated, for the first time, that acute BPA exposure (10^−9^ M) increased the duration of sustained ventricular arrhythmias in isolated female rat ventricular cardiomyocytes [45]. These arrhythmias were mediated through rapid Erα- and Erβ-dependent signaling mechanisms through a rapid modulation of Ca^2+^ handling, particularly via an increase in Ca^2+^ leakage from the sarcoplasmic reticulum. The previously mentioned effects were abolished when samples were pre-treated with an ER antagonist [45]. In the same sense, Belcher et al. showed that low nanomolar concentrations of BPA (0.001–1 nmol/L) and estrogen (17β-estradiol or E2) could sex-specifically alter estrogen-signaling in cultured adult rodent cardiomyocytes [46]. Two years later Yan et al. showed that acute BPA exposure, alone or combined with 17β-estradiol (E2), induces a double-edged effect in female rat hearts with ischemia–reperfusion (IR) injury. The authors showed that BPA (1 nmol/L) confers a protective effect against infarction, and impairs ventricular arrhythmia after IR injury [47]. In the same year, 2013, Gao et al. performed a study to elucidate the signaling mechanisms underlying the rapid impact of BPA on myocyte Ca^2+^ handling and arrhythmogenesis in female rat ventricular myocytes. The study shows that BPA (1 nmol/L) activates two parallel signaling pathways, the cAMP/PKA pathway, and the PLC/IP3/Ca^2+^/CAMKII pathway, which selectively impact two key Ca^2+^ handling proteins, ryanodine receptors and phospholamban (PLB) [48].

In 2019, Pinto et al., using cell lines, showed that BPA, bisphenol AF (BPAF) and bisphenol C (BPC) were agonists with different potencies for the three zebrafish estrogen receptors [49].

On the other hand, Ramadan et al. used neonatal cardiomyocytes to analyze the effect of BPA exposure [50]. These neonatal populations are more vulnerable to EDCs, so the study of these more vulnerable populations is of utmost importance, to be able to assess the effects of these compounds [51]. The authors exposed the cardiomyocytes to a wide range of BPAs that mimic environmental, clinical, and supraphysiological levels, and showed a reduction in spontaneous beating rate, an increase in heart rate variability (HRV), a reduction in the Ca^2+^ transient amplitudes, and prolongation of the Ca^2+^ transient upstroke and the duration time [50]. BPA exposure (1–100 µmol/L) also reduces the Ca^2+^ transient rise time and decreases the Ca^2+^ transient amplitude of hiPSC-CM in a dose-dependent manner [41].

Additionally, using mouse embryonic stem cells (ESCs) line R1, derived from 129 mouse strains that are differentiated in cardiomyocytes, Zhou et al. showed, in 2020, that individual and combined exposure to 10 ng/mL of BPA and 100 ng/mL of perfluorooctane sulfonate (PFOS) during embryonic stem cell differentiation could enlarge cardiomyocyte size, increase collagen expression, and damage mitochondria. In summary, combined exposure to PFOS and BPA could lead to adverse effects on heart development, and the interaction between PFOS and BPA may affect the rat fetal heart [52].

In EC, exposure to BPA (0–10 µmol/L) for 24 h increased the necroptosis/apoptosis ratio, the expression of Rat Receptor Interacting Protein 3 (RIP3), and CamKII activation. Moreover, the application of necrostatin-1, an inhibitor of necroptosis, improved BPA-induced cardiac dysfunction and prevented the inflammatory and hemorrhagic response in mice. In conclusion, these authors demonstrated that BPA activates the RIP 3-CamKII necroptotic pathway, leading to endothelial cell death. This mechanism may also be involved in heart failure, as the endothelial barrier loses its function; this leads to the weakening of the vascular wall of the coronary arteries in a hypertensive condition, causing ventricular hemorrhages and cardiac and pulmonary congestion [53].

In summary, it seems clear that BPA exposure induces electrical changes in cardiac muscle cells and vascular SMC, with L-type Ca^2+^ channels and voltage-dependent K^+^ channels being the most affected by BPA (Table 1). Thus, we can say that BPA inhibits Ca^2+^ channels and activates K^+^ channels, can act as a negative inotropic agent in cardiomyocytes, and may also have a negative chronotopic effect on both SMC and cardiomyocytes. Furthermore, there was also a clear association between exposure to BPA and alterations in Ca^2+^ handling, and in some parameters that promote oxidative stress, such as NO. Thus, and although much remains to be revealed about the mechanistic effects of BPA on the cardiovascular system, the association between the increase in cardiovascular pathologies, such as arrhythmias, and exposure to BPA seems clear.

### 4.2. Ex Vivo Studies

A few ex vivo studies have shown exposure to BPA in the cardiovascular system. The first ex vivo study was performed in rat atria by Pant et al. in 2011 [55]. The authors analyzed the direct action of BPA (0.1–100 μmol/L) on rat atria and demonstrated that BPA decreases the contractility of beating atria and decreases the rate and force of atrial contractions due to the activation of the NO–guanylyl cyclase pathway [55]. The Posnack group shows that exposure to higher concentrations of BPA (0.1–100 µmol/L) could decrease the rate and force of contractility and cardiac conduction velocity in the hearts of female rats [30] and, to a lesser extent, in the male heart [54]. More specifically, in 2014, it was first demonstrated, in whole hearts of adult female rats, that exposure to BPA adversely affected cardiac electrical conduction in a concentration-dependent manner. In this way, the authors showed that BPA exposure, after acute exposure (≤15 min), results in atrioventricular conduction delay, confirmed by the longer PR segment times and by the decrease in epicardial conduction. Exposure to the highest concentration of BPA resulted in longer QRS breaks and softened heartbeats, resulting in a complete blockage of the heart [30]. In 2015, the Posnack group observed, in female hearts, that during sinus rhythm, BPA exposure decreased left ventricular pressure and contractility activity (inotropic effect) in a dose-dependent manner. In male hearts, BPA exposure also modulated contractile performance, but to a lesser extent. Moreover, the study also showed that BPA exposure (0.001–100 µmol/L) modulated Ca^2+^ handling by reducing diastolic and systolic Ca^2+^ [54]. Therefore, the authors concluded that if these results were transposed to live experiments (human exposure), individuals, after chronic exposure, would be expected to show cardiac conduction abnormalities (i.e., bundle branch block, bradycardia, or arrhythmia).

In 2018, Feiteiro et al. performed a study in an organ bath to observe the vasorelaxant effect of BPA in rat aorta rings devoid of endothelium with noradrenaline (1 µmol/L) and potassium chloride (60 mmol/L). Afterward, cumulative concentrations of BPA (0.001–100 µmol/L) were administered to aortic rings, and it was observed that BPA induces rapid and concentration-dependent relaxation. In summary, the authors suggested that BPA inhibits the L-type Ca^2+^ channels, resulting in the relaxation of vascular smooth muscle. This non-genomic effect is similar to that observed for estradiol and other sex hormones in the same samples [37]. Thus, the authors proved that BPA also modulates the regulation of the vascular smooth muscle, which is essential for vasoreactivity, and may be involved in some cardiovascular diseases such as hypertension and coronary artery disease.

More recently, in 2021, Filice et al. performed ex vivo experiments on excised goldfish hearts. This research group showed that BPA affects the fish heart by inducing time- and concentration-dependent damage. First, they observed that the spontaneous heart rate was unaffected by BPA at 10 µmol/L, but for 25 µmol/L, a significant decrease was verified; this suggests a concentration-dependent chronotropic effect. Moreover, this previous response was also time-dependent, since the effect on animals exposed for 10 days was more pronounced than on those who were exposed for 4 days. In the hearts of goldfish treated with 10 µmol/L BPA, those exposed to 25 µmol/L required a higher preload pressure to achieve the physiological baseline cardiac output, leading the authors to suspect a detrimental effect of the BPA on basal performance [56].

In summary, the ex vivo experiments seem to corroborate the in vitro experiments, especially regarding the association between exposure to BPA and the development of cardiac arrhythmias and hypertension.

### 4.3. In Vivo Studies

Only a few in vivo mammalian studies have been performed regarding the effect of this EDC on the cardiovascular system. These studies were conducted on rodent and fish models. To simplify the understanding of the studies, we will first address all the studies on rats, chronologically and then those on fish, also chronologically.

The first study performed in vivo was in 2013, where Patel et al. demonstrated, in mice, that long-term exposure to BPA causes increased protein expression of DNMT3a (DNA methyltransferase 3a), and changes in patterns of protein expression and cardiac structure and function, as well as BP [57]. The authors also found a reduction in kidney weight, which might suggest that early exposure to BPA may impair kidney development in male mice. Moreover, the authors also identified differences between male and female rats after BPA exposure, such as concentric remodulation in males, and increased diastolic BP in all females. Some proteins that are essential for Ca^2+^ homeostasis, such as sodium Ca^2+^ exchanger-1, PLB, phospho-PLB, and calsequestrin 2, were changed in terms of quantification. Alterations in their expression support increased Ca^2+^ mobility in males and reduced Ca^2+^ mobility in females, becoming evidence of cardiac function changes. Finally, DNMT3a expression was increased in all BPA males and females who were given 0.5 µg/kg/day of BPA, and reduced in those females who received 200 µg/kg/day. These changes are suggestive of BPA-directed alterations detected in reproductive tissues and are also targeted in heart tissues [57].

In the next year, Saura et al. used CD1 mice orally administered BPA in their drinking water (4 nmol/L to 400 μmol/L). The authors showed that BPA induces high BP through Ang-II-mediated CaMKII-α uncoupling of eNOS and impairs carotid relaxation in mice. These data suggested that Ang-II-induced activation of CaMKII-α can play a key role in the endothelium dysfunction induced by BPA [58]. In the same year, Kim et al. investigated whether BPA (50 μg/kg body weight/day—12 weeks) would induce atherosclerosis, and; for this, the authors used an animal model of atherosclerosis, apolipoprotein E knockout (ApoE−/−) mice. The data suggested that an increase in non-HDL cholesterol levels may be a major contributing factor to BPA-induced atherosclerosis. The study also showed that expression of TNF-α and IL-6 in the aorta increased, but the serum levels of those inflammatory cytokines, which are the main inductor for atherosclerosis, were not changed [59].

In 2015, BP was studied by Belcher et al. and they showed that exposure to BPA resulted in decreased systolic and mean atrial pressures (MAP) in both male and female mice. Moreover, males exposed to BPA above 5 µg/kg/d presented a significant decrease in systolic BP and MAP, and in female rats, a significant decrease was only observed from the highest BPA exposure group, 300 µg/kg/d. Furthermore, in the same study, changes in the composition of the extracellular matrix of collagen were observed; these consisted of an accumulation of collagen in the heart, which may induce cardiac remodeling and abnormal fibrosis. The authors also performed transcriptome analysis and showed that BPA exposure can induce sex-specific alterations in gene expression, which indicated dysregulation of the collagen extracellular matrix and altered lipid metabolism of the rat heart. Thus, this study allowed us to conclude that BPA presents negative effects at the cardiac level, especially in response to cardiac ischemia [60].

In addition to the effects of BPA on Ca^2+^ handling, this compound may also have effects on some parameters of oxidative stress, such as NO. With regard to this, in 2015, the research group of Aboul-Ezz et al. studied the effect of BPA exposure, in rats that received a daily oral administration of BPA (25 mg/kg for 6 weeks and 10 mg/kg for 6 and 10 weeks), on the NO levels of male albino rats. The adverse effects of BPA on rat hearts were mainly due to the production of reactive oxygen species. In addition, it is postulated that the decreased level of NO and reduction in antioxidant defenses of the heart may result in vasoconstriction, which may lead to a decreased blood supply to the cardiac tissue and, ultimately, to a state of myocardial ischemia [61].

In the same year, 2015, Patel et al. performed a study to analyze whether BPA exposure (25 ng/mL^−5^ µg BPA/kg BW/day) is involved in cardiovascular remodeling. For this, C57bl/6n male mice were chronically exposed to BPA and myocardial infarction was induced in the mice. The data showed that chronic BPA exposure reduces remodeling after myocardial infarction by increasing monocyte and macrophage inflammation and reducing myofibroblast repair function [62].

Concerning the effect of BPA on hypertrophy in the NCTR Sprague Dawley rat, the first study to analyze the BPA effect in vivo was performed by Gear et al. in 2017 [63]. Rodent progressive cardiomyopathy is a common background lesion of undetermined etiology, and even though it occurs in both genders, it affects mainly males. This injury is suspected to arise from a localized microvascular dysfunction, and the resulting lesions phenotypically progress from minor to extensive focal mononuclear cell infiltration, myocyte degeneration, and fibrosis [64]. The research work of Gear et al. observed the cardiomyopathy-like lesions in the sections used for the characterization of left ventricular wall thickness and fibrosis. In this study on female rats treated with BPA or EE at 21 days of age (PND21), cardiomyopathy incidence was increased compared to control females, and a significant increase in severity was found for BPA and EE groups which received 2.5, 250, or 25,000 µg of these compounds. When the researchers observed the PND90 at 6 months, 100% of control-sample males and females had cardiomyopathy from both the stop dose and the continuous dose. Remarkably, the greatest morphometric effects observed were due to the fact that the duration of the treatment caused changes in body weight and the accumulation of cardiac collagen. Exposure to BPA caused an increase in the incidence and severity of progressive cardiomyopathy in female rats at 21 days, and increased the severity of cardiomyopathy in both sexes at 90 days [63].

In the same year, Klint et al. used juvenile female Fischer 344 rats to study BPA exposure (5, 50, and 500 μg BPA/kg bodyweight/day) in the cardiovascular function markers and fructose in in vivo cardiac tissues. The markers analyzed were vascular endothelial growth factor (VEGF), eNOS, and angiotensin I-converting enzyme (ACE1), which are known as estrogen-responsive genes in cardiovascular cells and tissues. VEGF exerts its function by targeting VEGFR2 (VEGF receptor 2), in EC. The oral low-dose BPA exposure of rats from pre-adolescence to adulthood up-regulated the expression of genes that control angiogenesis (Vegf and Vegfr2), a gene related to vasoconstriction (Ace1), and a gene related to endothelial dysfunction (eNos) [65].

In 2018, Sui et al. performed a study to analyze the effect of perinatal BPA exposure on atherosclerosis development in offspring. To this end, they developed a mouse model—PXR-humanized apolipoprotein E-deficient (huPXR•ApoE−/−)—to study BPA’s atherogenic effect. The data showed that perinatal BPA exposure (50 mg/kg) impaired atherosclerosis in adult male huPXR•ApoE−/− offspring but had no effects on their control newborn rats. The BPA perinatal exposure did not modify the plasma lipid levels; nevertheless, it increased aortic and atherosclerotic lesional CD36 expression, potentially through pregnane X receptor-dependent epigenetic regulation [66].

In 2019, Bruno et al. demonstrated that exposure to high doses of BPA increases the risk of female mice suffering from myocarditis, an inflammatory heart disease, with an inverted-U dose–effect curve. The data also showed that exposure to BPA significantly increased inflammatory mediators such as CD4^+^ T cells, IFNγ, IL-17A, TLR4, caspase-1, and IL-1β in the heart. The increase in cardiac fibrosis compared to the controls was also shown. To analyze the effect of BPA in cardiac remodeling, the effect of BPA in the mast cells was also analyzed, and an increase in the number and degranulation of these cells was observed, mainly in the pericardium. In summary, it was demonstrated that BPA exposure (0.5, 5, and 50 µg BPA/kg body weight) increases the risk of viral pericarditis, an inflammation of the pericardial layers, due to mast cell degranulation [67].

More recently, Reventun et al., in 2020, exposed mice to a more prolonged exposure; they were orally exposed to 4 × 10^−5^ mol/L of BPA in their drinking water for 16 weeks. The authors observed that BPA induces an increased heart rate, prolonged PQ interval, PR segment, and impaired cardiac contractility. Moreover, and as expected, BPA increased systolic and diastolic BP after 4 weeks, which was further elevated at 16 weeks [53]. In the same year, Zhou et al. demonstrated the effect of the exposure of pregnant rats to BPA, PFOS, or their combination for 19 days on fetal hearts. The authors observed that BPA alone, and in combination with PFOS, induced an increase in septal thickness in the ventricular tissue, and also increased myocardial collagen content. Additionally, these in vivo experiments’ results were confirmed by in vitro experiments, and the thickening of the ventricular septum may be related to the effects of this mixture exposure on mitochondrial metabolism [52].

The zebrafish (*Danio rerio*) model has been used on studies to assess cardiovascular development and function [68,69,70]. Indeed, although the zebrafish heart is composed of a single ventricle, the cardiac electrophysiologic system and characteristics are similar to that of a four-chambered vertebrate [70]. Furthermore, zebrafish have a high homology of thyroid hormone signaling with mammals, enabling extrapolation to human health effects and the extensive accumulated knowledge on their thyroid signaling pathways [71]. Moreover, the model is also very important to study environmental pollutants, mainly EDCs, since these models have three ERs (zfERα, zfERβ2, and zfERβ1) that are 50% homologous in their amino-acid sequence identity to the human ER [72]. Therefore, by studying the implications of exposure to EDCs on the cardiovascular system of this model, we can extrapolate the observed adverse outcomes to human health.

In a study carried out on zebrafish embryos, Cypher et al. analyzed the cardiovascular response during early development and discovered that it was altered by the presence of BPA and hypoxia (0.25, 1 and 5 mg/L and 1.0 mg O_2_/L). The results showed that all the cardiovascular parameters analyzed, except for venous diameter, reduced more during exposure to BPA and hypoxia together than to BPA and hypoxia alone. This joint effect was synergistically superior, and there was an interaction between both parameters. Thus, the authors demonstrated, for the first time, that BPA exposure modifies the cardiovascular system during hypoxia more so than during normoxia [73].

In the same year, 2015, in a study performed on adult zebrafish, Lombo et al. assessed the potential effects of BPA in paternal exposure on offspring development. Adult zebrafish males were exposed to BPA during spermatogenesis and reproduced with control females. The results demonstrated that exposure to BPA increases the rate of heart failure in the progeny to F2 and decreases the gene expression of cardiac development in F1 embryos. Furthermore, it was also observed that after exposure to 2000 µg/L BPA, an increased percentage of cardiac malformations occurred in future generations (F1 and F2), such as cardiac edema and incorrect looping, and showed disorganized heart walls [74]. Thus, this work allowed us to conclude, for the first time, that exposure to adult males may promote adverse cardiovascular effects in the following two generations.

Three years later, in 2018, Cypher et al. developed the study discussed above, and observed that co-exposure to BPA (0.001–100 µg/L) and hypoxia could interfere with cardiovascular function, and vascular parameters were the most affected, particularly at lower BPA concentrations. BPA and oxygen concentration interacted to affect vascular parameters, particularly arterial red-blood-cell velocity [75]. In the same year, another study investigated the estrogenic responses and estrogen receptor signaling mechanisms by which BPA and its metabolite 4-Methyl-2,4-bis(p-hydroxyphenyl)pent-1-ene (MBP) act. The data showed that MBP in zebrafish has an effect several orders of magnitude greater than the effect observed for BPA. Furthermore, it was also demonstrated that the development of atrioventricular valves and bulbus arteriosus are major constituents in the heart, both for BPA and its metabolite. Estrogenic signal transduction for both bisphenols is mediated via an estrogen receptor 1-dependent pathway [76]. The following year, in 2019, the same research group demonstrated that exposure to BPA (100 and 1000 µg/L) and MBP (2.5 and 25 µg/L) activates the Estrogen Response Element (ERE) that acts primarily on heart valves. The integrity of the heart valves after exposure was compromised, with extra-cellular matrix collagen deficiency being the main reason for this, resulting in a modification of vascular function (reduced ventricular beat rate and blood flow) [77].

On the other hand, Pinto et al. demonstrated the estrogenic activity of BPA, BPAF, and BPC in zebrafish, and showed zfERα selectivity via the activation of the GFP reporter in the heart valves of zebrafish larvae. The authors also concluded that BPAF and BPC have a bigger affinity for zebrafish receptors than that observed by BPA [49].

Later, the same research group, Lombó and Herráez, observed that exposure to BPA during the early stages of development seriously affects the development of the heart. The exposure of BPA (2000 and 4000 µg/L) on embryos led to changes in cardiac phenotype; induced overexpression of hand2, a crucial factor for cardiomyocyte differentiation; increased the ER expression (esr2b); promoted an overexpression of histone acetyltransferase (kat6a); and also caused an increase in histone acetylation, estrogenic and epigenetic mechanisms; the latter are closely related, might act in synergy, and could be responsible for the upregulation observed in the transcription factor hand2, crucial for cardiac formation [78]. In the last year, in 2021, the same authors investigated the underlying molecular mechanisms of paternal exposure to BPA (100 and 2000 μg/L) to induce long-term effects on F1 cardiogenesis. The results showed that male exposure induces an increase in sperm histone acetylation (which is inherited by the F1) and, thus, alters the chromatin structure of the genes essential for heart development and those of the HAT in charge of maintaining the profile. Furthermore, when F1 embryos obtained from BPA-exposed males were treated with EGCG, histone acetylation levels were restored (which prevent ER overexpression and transcription factors) and, thus, reduced the likelihood of heart disease [79].

More recently, in 2022, Ji et al. studied the developmental vascular toxicity of BPA (0.25–12 mg L^−1^) and three predominant substitutes (BPF, BPS, and BPAF) in zebrafish embryos. They demonstrated that all drugs induce adverse effects on early vascular development. Moreover, BPAF showed the highest vascular toxicity, followed by BPF and BPA, while BPS exhibited the weakest toxicity [80].

Concerning the experiments with other fishes, a study on the role of BPA on tissue stress was performed by Filice et al. in the goldfish heart, by analyzing stress and pro-apoptotic markers, such as HSPs, Bax, and Cytochrome c, in cardiac extracts. The results showed that despite the unchanged expression of Hsp70 and Hsp90 in the presence of BPA 10 µmol/L, it significantly decreased in animals exposed to 25 µmol/L for the same period. However, they revealed the hypothesis that apoptosis is activated at low concentrations of the EDC; this is explained by the increased levels of the pro-apoptotic markers Bax and Cytochrome c which were detected after exposure at 10 µmol/L of BPA, but not at 25 µmol/L. This same study showed unchanged lipid peroxidation levels, increased OMP (oxidatively modified proteins) levels, and increased SOD (superoxide dismutase) activity in goldfish hearts exposed to 10 µmol/L, which suggests that in the presence of low BPA concentrations, increased SOD activity may contribute to counteracting lipid peroxidation. On the other hand, at high BPA concentrations, not only may lipid peroxidation and OMP decrease, but so may the activity of the antioxidant enzyme [56]. In 2022, Schönemann et al. showed that 7 days of exposure to 10 μg/L of the BPA metabolite, MBP, altered the liver proteome of male *Cyprinodon variegatus* fish. Furthermore, MBP enhanced ribosomal activity, protein synthesis, and transport, with upregulation of 91% of the ribosome-related proteins, and 12 proteins whose expression is regulated by ERE. Moreover, the acidic protein (WAP) was the protein most affected by MBP exposure, indicating that WAP may be a good new biomarker for xenoestrogens [81].

In conclusion, in vivo studies corroborate in vitro and ex vivo studies, and demonstrate that BPA and its metabolites can cause alterations in the cardiovascular system, leading to various pathologies such as higher BP, arrhythmias, atherosclerosis, endothelial dysfunction, cardiac ischemia, myocardial infarction, cardiomyopathy, myocarditis, congenital heart defects or cardiac anomalies, which may develop up to the second generation (F2). Further studies are, therefore, required to determine the exact mechanisms by which this bisphenol induces adverse cardiovascular effects. Summaries of the disruptive effects of BPA in the animal in vitro, ex vivo, and in vivo studies can be seen in Table 1, Table 2 and Table 3, respectively.

## 5. Effects of BPA on Humans

Several studies have shown that there is an increased risk of premature CVDs positively associated with environmental factors, such as exposure to EDCs [31]. Environmental exposure to BPA appears to be harmful to human health; however, there are still few studies assessing the toxicity of this EDC in humans. This poses some concern because BPA has a potentially disruptive effect, but also because exposure to this compound is ubiquitous [82]. Additionally, BPA’s ability to cause adverse effects on humans is well documented in epidemiological studies, supporting a positive association between higher exposure to BPA and an increased risk of CVDs or risk factors for them. Therefore, this section will address the main disrupting effects of BPA described in humans through in vitro studies (namely on ion channels and electrophysiology, Ca^2+^ handling, vascular endothelium, and pregnancy exposome) and the epidemiological evidence supporting a relationship between BPA and CVDs (including risk factors and pregnancy exposure) (please see below).

### 5.1. In Vitro Studies

#### 5.1.1. Effects of BPA on Ion Channels and Electrophysiology

Chemical exposure to BPA has been associated with changes in cardiac excitability, notably through changes in HRV and/or electrical conduction [27]. As recently reviewed by Cooper and Posnack [27] and Ramadan et al. [6] several studies have shown that BPA has an inhibitory effect on individual ion channels, with an important role in the regulation of cardiac action potentials.

As heart toxicity may derive from modified cardiac electrophysiology, O’Reilly et al., in 2012, investigated the interaction between BPA and the human Nav1.5 channel (the predominant voltage-gated Na^+^ channel subtype expressed in the human heart and responsible for the action potential upstroke). The electrophysiology results in the HEK-transfected cell line showed that BPA (1–100 µmol/L) blocks the channel (K_d_ = 25.4 ± 1.3 µmol/L). Docking predictions suggested that BPA-induced blockage involves the local anesthetic receptor and may enter the closed-state pore via membrane-located side fenestrations, possibly via a cavity delimited by F1760 and contiguous with the DIII–IV pore fenestration [43]. In cardiac tissue, this effect on Na^+^ channel currents will reduce the rate of depolarization and slow cardiac conduction velocity [6]. According to these authors, Prudencio et al. also recently reported, in 2021, that BPA has a half-maximal inhibitory concentration (IC_50_) of 55.3 µmol/L and 23.6 µmol/L BPA for fast/peak and late Na^+^ channel currents, respectively, using the same cell type [42]. In the same year, Ae Hyun et al. also demonstrated that BPA (1–100 µmol/L) significantly inhibited sodium current (I_Na_) channels (IC_50_ = 56.5 µmol/L) [41]. Using both optical and microelectrode array methodologies, the authors suggested that BPA exposure altered cardiac function in human-induced pluripotent stem-cell-derived cardiomyocytes (hiPSC-CMs), as a slowing of the action potential upstroke (1–100 µmol/L BPA) and a reduction in the action potential amplitude (30–100 µmol/L BPA) were observed. These alterations may be due to the inhibition of I_Na_ channels [41]. As recently reviewed by Horváth et al., the late sodium currents’ (I_Na_, late) inhibitors are potential antiarrhythmic agents. Increased late I_Na_ seems to play an important pathophysiological role in cardiac diseases, including rhythm disorders. Regarding the BPA-induced blockade, as the late I_Na_ channels are active during the plateau action potential phase, it is expected that this EDC shortens the repolarization time and, in turn, reduces the I_Ca_ channels [44].

In this sense, some studies have also shown that BPA can inhibit I_Ca_. In 2013, Deutschmann et al. reported that BPA (1–100 µmol/L) acts as a potent blocker of voltage-activated Ca^2+^ channels. Briefly, the authors determined the mechanisms of blockage and the structural elements of BPA essential for its action, and verified that BPA rapidly and reversibly inhibited the recombinant human R-type Ca^2+^ channels expressed in HEK 293 cells. BPA binding to the channel occured in the extracellular part (outside the pore formation region), not involving intracellular signaling pathways. Moreover, this binding was voltage independent and did not affect channel gating, indicating that binding occurs with the channel in its resting state [38]. On the other hand, as T-type Ca^2+^ channels are important regulatory elements in the cardiovascular system, in the same year, Michaela et al. also performed electrophysiological studies to evaluate the effects of BPA on these channels in HEK 293 cells. The authors observed a concentration-dependent inhibition of T-type Ca^2+^ channels; for nanomolar concentrations of BPA, they observed inhibition of the channels (order of efficiency: CaV_3.2_ ≥ CaV_3.1_ > CaV_3.3_) without affecting the voltage dependence and kinetics of channel gating. However, BPA at micromolar concentrations accelerated the current-decay kinetics, shifted the voltage dependence of steady-state inactivation to more negative values, and inhibited the current amplitudes. Thus, the authors suggested that BPA (1–100 μmol/L) appears to act as a modifier of channel gating and directly plugs the pores of the conductive channel at high concentrations. The concentration range in which BPA-induced inhibition was observed corresponds to concentrations detected in human fluids; therefore, it may be relevant for the evaluation of the effects of this EDC on cardiovascular health, not least because T-type Ca^2+^ channels are expressed in nodal and conduction cells [39]. Regarding L-type Ca^2+^ channels, in 2021, Ae Hyun et al. also demonstrated that BPA dose-dependently inhibited I_Ca_ channels in hiPSC-CMs (IC_50_ = 6.9 µmol/L). These results seem to suggest that BPA can lead to cardiac dysfunction and cardiac risk factors (e.g., arrhythmias) [41]. In this context, it is expected that the effects of BPA on cardiac physiology are mediated by a Ca^2+^-dependent mechanism, given the importance that these channels have both for cardiac excitability and contractility. Moreover, due to the inhibitory effects that BPA exerts on L-type Ca^2+^ channels, and given the role of these channels in the plateau phase of action potentials, it is expected that BPA can also reduce the duration of the action potential. Concordantly, Ae Hyun et al. also revealed that acute exposure to BPA can shorten the optic action potential (10–100 µmol/L) [41]. Moreover, BPA can also exert effects on other ion channels; for example, Asano et al. reported that 100 µmo/L of BPA increases the large-conductance Ca^2+^/voltage-sensitive K^+^ channel (Maxi-K) current in human coronary artery SMC [35].

In summary, and taken together, the literature agrees that BPA has a dose-dependent monotonic effect on voltage-gated Na^+^ and Ca^2+^ channels [27,83], impairing cardiac electrophysiology (please see Table 4). Scientific evidence agrees that BPA has effects on Ca^2+^ currents, directly impairing cardiac automaticity and electrical conduction. However, further studies are needed to clarify whether these effects are sex-specific and what the dose–response relationships are with the application of BPA alone or in combination with other bisphenols. Furthermore, it is still important to clarify whether the effects induced by BPA are direct or involve other intracellular signaling pathways, as well as to unveil what role this EDC plays in cardiac development [6].

#### 5.1.2. Effects of BPA on Ca^2+^ Handling

Some research on rodent models has demonstrated the effects that BPA exposure has on intracellular Ca^2+^ handling, contractility, and relaxation, as previously described in Section 4. Most studies associate these effects with the inhibition of ionic currents and/or phosphorylation of key regulatory proteins induced by BPA [27], not least because BPA can inhibit Ca^2+^ ion influx through interaction with voltage-gated Ca^2+^ channels. Thus, and since cardiomyocyte contractility is proportional to the magnitude of this slow inward current, BPA may act as a negative inotropic agent [27,84].

In humans, BPA exposure has also been associated with alterations in both Ca^2+^ release and/or sequestration back to the sarcoplasmic reticulum [85]. With regard to this, Ae Hyun et al. demonstrated that BPA dose-dependently inhibited I_Ca_ channels, Ca^2+^ transients, and contraction in hiPSC-CMs. The results showed that acute exposure to BPA (1–100 µmol/L) in a dose-dependent manner slows the Ca^2+^ transient rise time and decreases the Ca^2+^ transient amplitude of hiPSC-CMs [41]. In another perspective, Cheng et al. analyzed how BPA at low doses of BPA (equivalents to human internal exposure levels) could induce cardiac hypertrophy via the calcineurin (CnAβ)-dynamin-related protein 1 (DRP1) signaling pathway by disrupting Ca^2+^ homeostasis. Using human embryonic stem-cell-derived cardiomyocytes (XX and XY karyotypes), the authors discovered that BPA (8 ng/mL) significantly elevated hypertrophic-related mRNA expression levels, enhanced cellular area, and reduced ATP supplementation, evidencing a hypertrophic cardiomyocyte phenotype in vitro. Additionally, BPA-induced excessive fission was promoted via CnAβ-mediated dephosphorylation of DRP1. At the molecular level, the increase in cytosolic Ca^2+^ levels due to low doses of BPA could discriminate between karyotyped-derived cardiomyocytes. Thus, because the results are more prominent in XX-karyotyped cells, these results are suggestive that there exists a potential BPA-induced sex-specific hypertrophic risk in terms of abnormal mitochondrial fission and ATP production through the impairment of CnAβ-DRP1 signaling [86].

In summary, to our knowledge, these were the only two studies in human cells that evaluated the effects of BPA on Ca^2+^ handling (please see Table 4), and there are also no in vivo studies that address the effects of BPA on cardiac contractility. Nevertheless, the literature is in agreement with studies in rodent models, suggesting that human exposure to BPA also has disruptive effects on intracellular Ca^2+^ handling. Further studies are needed to clarify the role of this EDC on cardiac contractility at this point.

#### 5.1.3. Effects on Vascular Endothelium

Several investigations have suggested that urinary BPA levels are associated with the pathogenesis of age-related pathologies (where CVDs is included). Vascular endothelium dysfunction may be implicated in CVDs [87], including atherosclerosis. The literature describes that there exists an association between elevated exposure to BPA and CVDs; however, little is known about the effects of BPA on the human endothelium. With regard to this, Andersson and Brittebo (2012) were the first authors to investigate the effects of BPA (0.1 nmol/L–1 μmol/L) on selected biomarkers of endothelial dysfunction, inflammation, and angiogenesis in human umbilical vein endothelial cells (HUVECs). The authors demonstrated that BPA (≤1 μmol/L) increased the mRNA expression of the proangiogenic genes (VEGFR-2, VEGF-A, eNOS, and Cx43) and increased NO production in HUVECs. Moreover, the results also showed that BPA increased the expression of phosphorylated eNOS and endothelial tube formation in the cells. Thus, the authors suggested that exposure to BPA has direct proangiogenic effects on human primary EC in vitro and, more importantly, human endothelium may be an important target for BPA [88]. Ribeiro-Varandas et al. also demonstrated that BPA at plasma concentrations (0.5–10 ng/mL) induces aneugenic effects on EC. The authors found alterations in micronucleus formation and cell division processes (leading to mitotic abnormalities), as well as positive regulation of the genes of encoded proteins associated with chromosomal segregation [89]. In the following year (2014), the same authors also reported that BPA (10 ng/mL and 1 µg/mL) impairs transcription and decreases viability in aging vascular EC. The authors suggested that BPA is associated with the etiology of age-related human pathologies, such as atherosclerosis, by interfering with senescence in primary vascular EC [90]. Kim et al. also demonstrated that BPA (0.1–10 nmol/L) appears to be involved in accelerating atherosclerosis but found no changes in HUVEC proliferation or migration [59].

Taken together, these findings suggest that BPA exposure may induce endothelial dysfunction, promoting the development of age-related diseases such as atherosclerosis (please see Table 4). Further studies are needed to clarify the adverse effects adjacent to BPA exposure and the mechanisms of toxicity by which the vascular endothelium is impaired. In this way, better targeting in clinical practice will be achieved.

#### 5.1.4. Effects in Pregnancy Exposome

In recent years, there has been increased interest in the endocrine-disrupting effects of BPA on human pregnancy and fetal development. As recently reviewed by Lorigo and Cairrao, several studies have reported the presence of BPA in various biological matrices important in pregnancy (including maternal blood and urine, amniotic liquid, placenta, umbilical cord blood, breast milk, and human colostrum) [51]. Usually, detected concentrations of BPA are higher in urine and, therefore, urine is preferentially the most-used biological sample (not least because BPA is a non-persistent compound) [6,82]. Nevertheless, different analytical techniques have been performed to detect BPA in human samples [7].

Worryingly, concerning pregnancy, studies have shown that BPA can not only penetrate, but also accumulate, in the human placenta, with studies even demonstrating higher levels of this EDC in the placenta than in maternal plasma [6]. Consequently, this maternal–fetal transfer may be one of the causes of BPA-induced cardiovascular disorders in adulthood [51].

Indeed, BPA is one of the most-studied EDCs, resulting in epigenetic disruption, including changes in DNA methylation, acetylation, genomic imprinting, and modifications in the expression of microRNAs and non-coding RNAs [51]. These changes are particularly important in those who are pregnant, a susceptible populational group, as the fetus has a high rate of DNA synthesis. In 2014, Nahar et al. investigated the hypothesis that in utero exposure to BPA influences the expression and epigenetic regulation of phase I and II xenobiotic-metabolizing enzymes (XME) genes during development. The authors found an association of higher levels of BPA (35.4–56.1 ng/g) with significantly reduced expression of XME genes, and with increased site-specific methylation at COMT and increased average methylation at SULT2A1 promoters [91]. Later, in 2018, Montrose et al. also investigated whether maternal exposure to BPA (0.57 and 0.78 ng/mL) in the first trimester of pregnancy is associated with infant cord blood DNA methylation. The authors found decreases in the methylation of imprinted (H19, IGF2) and unprinted (PPARA, ESR1) genes and of repetitive element LINE-1 (long interspersed nuclear element-1 or L1), with increasing BPA concentrations [92]. Overall, it seems that BPA accumulation in the maternal placenta is responsible for global methylation, which may lead to fetal growth retardation [93,94].

In addition to BPA-induced epigenetic changes, studies are reporting a direct effect on pregnancy physiology. In 2010, Mørck et al. conducted studies in the BeWo trophoblast cell line, placental explant cultures, placental perfusions, and skin diffusion models, all of human origin, to assess the effects of BPA exposure during pregnancy. The authors demonstrated that there is BPA cytotoxicity in these cells (EC_50_ = 100–125 µmol/L). BPA exposure (1 nmol/L) significantly increased β-hCG secretion and caspase-3 expression in placental explants. A rapid transfer of this EDC through the term placentae and the BeWo cell monolayer was also observed, as well as transdermal transport. Thus, these results seem to indicate that placental transfer of BPA (1 nmol/L) to the fetus occurs, with potentially adverse effects on both placental and fetal development [95]. Concordantly, and using the same cells, Ponniah et al. also demonstrated, in 2015, that BPA exposure (0–9.0 μmol/L) may have implications on placental trophoblasts during development, as this EDC induced trophoblast cell death under conditions of cellular stress [96]. Additionally, in the same year, Spagnoletti et al. evaluated the effect of BPA (1 × 10^−15^ to 1 × 10^−7^ mol/L) on the main physiological processes which characterize the extravillous trophoblast in human trophoblast cells HTR-8/SVneo. The results showed that BPA acts on these cells, altering key physiological processes in placenta development. Although the exact mechanism by which BPA acts on human trophoblasts needs further clarification, this study showed that there are reductions in the processes of cell migration and invasion as well as a differentiation of HTR-8/SVneo towards polyploidy via the process of endoreduplication induced by BPA [97]. More recently, in 2018, Basak et al. also demonstrated that low concentrations of BPA (1 nmol/L) can not only affect cellular growth and development and angiogenic activities, but also, concordantly with previous studies, induce alterations in DNA methylation of the stress response and down-regulation of angiogenic growth factors. These alterations were observed using HTR8/SVneo cells, during the first trimester of pregnancy [98].

Therefore, the mentioned studies are concordant in their statements that exposure to BPA plays a determinant role in pregnancy physiology. Additionally, exposure to BPA has also been associated with adverse placental outcomes v multiple mechanisms of action (MOAs) [99,100,101]. Consequently, BPA exposure may also induce several fetal and obstetric outcomes. Risk of increased pregnancy loss, longer pregnancies or preterm birth, metabolic dysfunction, altered somatometric parameters, and newborn weight are some of the demonstrated outcomes (please see reviews [100,102]).

In summary, all the studies agree that BPA exposure plays a determining role in pregnancy, and consequently, may increase susceptibility to CVDs in later life (please see Table 4). In addition to BPA-induced epigenetic changes, this EDC also directly affects the physiology of pregnancy, which, taken together, highlight the need for future studies to assess the risk of BPA-induced reproductive toxicity.

**Table 4:** Summaries of the disruptive effects of BPA in in vitro studies using human cell lines ^1^.

Topic	Studied Mechanism	Concentration	Type of Cells	Observed Effects	References
Ion channels and electrophysiology	Nav1.5 channels	1–100 µmol/L	HEK-transfected cell line	▪ BPA blockage of the channel (Kd = 25.4 ± 1.3 µmol/L); ▪ BPA-induced blockage involved the local anesthetic receptor and may have entered the closed-state pore via membrane-located side fenestrations.	[43]
	Nav1.5 channels	0.0–100 µmol/L	HEK-transfected cell line	▪ BPA had a half-maximal inhibitory concentration (IC_50_) of 55.3 µmol/L and 23.6 µmol/L BPA for fast/peak and late Na^+^ channel currents	[42]
	Nav1.5 channels	1–100 µmol/L	hiPSC-CMs	▪ BPA significantly inhibited Na^+^ current channels (IC_50_ = 56.5 µmol/L) ▪ BPA slowed the action potential upstroke (1–100 µmol/L) ▪ BPA reduced the action potential amplitude.	[41]
	Recombinant human R-type Ca^2+^ channels	1–100 µmol/L	HEK 293 cells	▪ BPA included rapid and reversible inhibition of the channels. ▪ BPA binding occured with the channel in its resting state, and in the extracellular part not involving intracellular signaling pathways.	[38]
	T-type Ca^2+^ channels	1–100 μmol/L	HEK 293 cells	▪ BPA appeared to act as a modifier of channel gating and directly plugged the pores of the conductive channel at high concentrations.	[39]
	L-type Ca^2+^ channels Cav1.2	1–100 µmol/L	hiPSC-CMs	▪ BPA dose-dependently inhibited Ca^2+^ current channels (IC_50_ = 6.9 µmol/L).	[41]
	Maxi-K channels	100 µmol/L	HCASMC	▪ BPA increased Maxi-K currents	[35]
Ca^2+^ handling	Ca^2+^ current channels, Ca^2+^ transients and contraction	1–100 µmol/L	hiPSC-CMs	▪ BPA in a dose-dependent manner slowed the Ca^2+^ transient rise time and decreased the Ca^2+^ transient amplitude	[41]
	Cardiac hypertrophy by disrupting Ca^2+^ homeostasis	8 ng/mL	Human embryonic stem-cell-derived cardiomyocytes	▪ BPA induced sex-specific hypertrophic risk in terms of abnormal mitochondrial fission and ATP production by impairing CnAβ-DRP1 signaling	[86]
Vascular endothelium	Endothelial dysfunction, inflammation, and angiogenesis	0.1–1 μmol/L	HUVECs	▪ BPA increased the mRNA expression of the proangiogenic genes and increased NO production ▪ BPA increased the expression of phosphorylated eNOS and endothelial tube formation	[88]
	Cell division and chromosomal segregation	0.5–10 ng/mL	HUVECs	▪ BPA at plasma concentrations induced aneugenic effects	[89]
	Senescence	10 ng/mL and 1 µg/mL	HUVECs	▪ BPA impaired transcription and decreased viability in aging vascular EC	[90]
	Accelerating atherosclerosis	0.1–10 nmol/L	HUVECs	▪ BPA appeared to be involved in accelerating atherosclerosis ▪ BPA does not altered the HUVEC proliferation or migration	[59]
Pregnancy exposome	Epigenetic disruption	35.4–56.1 ng/g	Human fetal liver samples	▪ Higher levels of BPA with XME genes significantly reduced expression and with increased site-specific methylation at COMT and increased average methylation at SULT2A1 promoters	[91]
		0.57 and 0.78 ng/mL	Maternal urine samples and Infant cord blood	▪ BPA decreased methylation of imprinted and unprinted genes and repetitive element LINE-1	[92]
	Pregnancy physiology	1 nmol/L	BeWo trophoblast cell line, placental explant cultures, placental perfusions, and skin diffusion models	▪ BPA induced cytotoxicity (EC_50_ = 100–125 µmol/L). ▪ BPA significantly increased β-hCG secretion and caspase-3 expression in placental explants.	[95]
		0, 0.09, 0.9, and 9.0 μmol/L	BeWo trophoblast cell line	▪ BPA induced trophoblast cell death under conditions of cellular stress	[96]
		1 × 10^−15^ to 1 × 10^−7^ mol/L	Human trophoblast cells HTR-8/SVneo	▪ BPA altered key physiological processes in placenta development	[97]
		1 nmol/L	Human trophoblast cells HTR-8/SVneo	▪ BPA induceed alterations in DNA methylation of stress response and down-regulation of angiogenic growth factors	[98]

^1^ Legend: BPA—bisphenol A; CnAβ—calcineurin; DRP1—dynamin-related protein 1; HCASMC—human coronary artery smooth muscle cells; HEK—human embryonic kidney; hiPSC-CMs–human-induced pluripotent stem-cell-derived cardiomyocytes; HUVECs—human umbilical vein endothelial cells; IC50—half-maximal inhibitory concentration; LINE-1—long interspersed nuclear element-1 or L1; Maxi-K—large conductance Ca^2+^/voltage-sensitive K^+^ channel; XME—xenobiotic-metabolizing enzymes.

### 5.2. Epidemiological Studies

**Figure 2:**
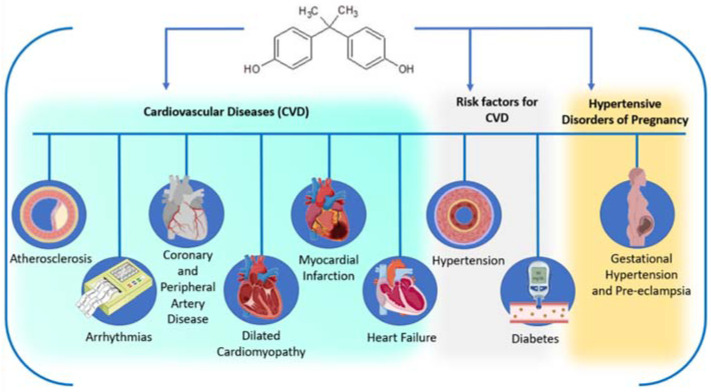
Summary representation of the endocrine-disrupting effects of bisphenol A on the cardiovascular system.

Several epidemiological investigations have been developed to assess the cardiotoxic effects of BPA. The evidence is consistent, reporting that there is an association between exposure to BPA and an increased prevalence of CVDs [103,104] including atherosclerosis [105], coronary artery disease (CAD) [104,106,107,108,109], peripheral artery disease (PAD) [110], dilated cardiomyopathy (DCM) [109], and myocardial infarction (MI) [104,111]; heart failure; and angina pectoris [104] and its risk factors, such as hypertension [112,113,114,115] and diabetes [103,108] (Figure 2). There is a multiplicity of different analytical techniques to quantify BPA concentrations in human tissues. However, due to their short half-lives (<24 h), the concentration of this EDC is measured preferentially in urine, the matrix where the concentration detected is higher (the concentration ranges from 1.6 to 946 μg/L) [51]. Regarding the association between BPA and adverse effects on the cardiovascular system, the maximum mean concentration detected was 4.66 ng/mL. Furthermore, exposure to BPA is also particularly harmful in more sensitive windows of development, such as pregnancy, which are, in these cases, related to the development of hypertensive disorders of pregnancy (HDPs). The serum range levels detected in pregnant women are from 0.2 to 20 ng/mL. and in placental tissue the concentration detected was much higher, at around 100 ng/g. In urine samples, the concentration ranged from 0.87 to 31.9 μg/L [51,116] for the pregnant women, and 1.65 ng/mL when there was an association between urinary BPA concentrations and CVDs (Table 5). With regard to this, epidemiological studies reporting the effects of BPA exposure and its association with CVDs and risk factors, as well as the implications of exposure during pregnancy, will be discussed in detail in the next two sections.

#### 5.2.1. Cardiovascular Diseases and Risk Factors

Several epidemiological studies have been carried out, mostly from a National Health and Nutrition Examination Survey (NHANES) dataset. The assessment of cardiovascular parameters was performed using urine samples from the participants for whom BPA concentrations were analyzed [13]. The first study was conducted in 2008 by Lang et al., who analyzed data from NHANES from 2003 to 2004 and showed that there is an association between CVDs and elevated urinary BPA levels (OR = 1.39), after adjusting for the confounding factors age and sex. The authors also found a relationship between higher BPA concentrations and diabetes (OR = 1.39) [103]. On the other hand, a relationship between BPA exposure and PAD and/or CAD was also assessed and found. With regard to this, Melzer et al., in 2010, also analyzed BPA concentrations with heart diseases by analyzing NHANES 2003–2006 data, separately between 2003/2004 and 2005/2006, and pooled. The results of urinary BPA concentrations were smaller for the 2005/2006 data (mean 1.79 ng/mL) than those from 2003/3004 (mean 2.49 ng/mL). After adjustment for confounding factors, the authors found that there was an association between higher BPA levels (2003/3004 data) with CAD (OR = 1.33), but the same was not observed with diabetes. Contrarily, when data were pooled, an association between higher BPA concentrations and these diseases was found (OR = 1.42 for CAD and OR = 1.24 for diabetes) [108]. In summary, the authors verified that higher levels of urinary BPA were cross-sectionally associated with heart disease in NHANES 2003–2004 and NHANES 2005–2006, independent of traditional risk factors. Thus, and due to BPA levels having previously been associated with CAD (which is an atherosclerotic disease), Lind et al. also evaluated, in 2011, a possible relationship between exposure to BPA and atherosclerosis in a cross-sectional study. The results showed that elevated levels of BPA were related to the echogenicity of the plaques, suggesting a role for BPA in atherosclerosis [105]. One year later, Melzer et al. continued their previous studies, using data for 10.8 years from the European Prospective Investigation of Cancer—Norfolk, UK. The authors showed that urinary BPA concentrations were low (median value, 1.3 ng/mL) and that an increased BPA concentration was associated with incident CAD, after adjustment for the confounding factors age, sex, and urinary creatinine (OR = 1.13) [106]; similar trends were obtained to the previous study by the same authors [108]. Additionally, in the same year, but in a different study, Melzer et al. also studied the possible association between BPA exposure with grades of severity of CAD on angiography [107] in 591 patients participating in The Metabonomics and Genomics in Coronary Artery Disease study in Cambridgeshire, UK. The authors found that there was a higher urinary concentration of BPA in patients with severe CAD compared to patients with normal coronary arteries (OR = 1.43; 95% CI = 1.03–1.98, *p* = 0.033). Furthermore, for the case of patients with intermediate disease, significant differences were also almost obtained (OR = 1.69; 95% CI = 0.98–2.94, *p* = 0.061). Thus, it can be concluded that BPA exposure was associated with the severity of angiography-defined coronary artery stenosis and may contribute to a future risk of developing CAD in the adult population [107]. Taken together, these findings highlight the need for further studies to accurately estimate the prospective exposure–response curve and, thus, establish the underlying mechanisms [106,107].

Additionally, in 2012, another study by Shankar et al. examined the relationship between urinary BPA levels and peripheral arterial disease (PAD) (which is a subclinical measure of atherosclerotic vascular disease and a strong independent risk factor for CVDs and mortality) in a nationally representative sample of U.S. adults [110]. This study, which was conducted with participants from NHANES 2003–2004, also showed that there is a positive association between increased levels of BPA with DBP, independent of traditional CVD risk factors (OR = 2.69; 95% CI = 1.02–7.09; *p* = 0.01) [110]. Although they cannot provide definitive conclusions, these results suggest that environmental exposure to BPA may promote the development of PAD [110]. Additionally, in 2015, Xiong et al. performed a case-control study evaluating the concentrations of BPA in patients with DCM, and demonstrated higher levels of BPA in DCM patients compared with the healthy group (6.9 ± 2.7 ng/mL vs. 3.8 ± 1.9 ng/mL, *p* < 0.001), suggesting that BPA exposure may promote the development of this pathology [109]. On the other hand, if a positive association between BPA exposure and CAD has been shown, this has not been evaluated in patients with type 2 diabetes (T2D). With regard to this, in 2019, Hu et al. evaluated this possible relationship through two nested case-control studies in two independent European cohorts (SURDIAGENE and ESTHER). The authors demonstrated the exposure to BPA in the two studies (ESTHER cohort: 31% and SURDIAGENE cohort: 38%). The meta-analysis of the results showed that in booth cohorts, there exists a positive association between MI and the detection of BPA in urine (OR = 1.97; 95% CI = 1.05–3.70, *p* = 0.04) [111]. More recently, in 2020, Cai et al. evaluated the relationship between urinary BPA levels and CVDs in the U.S. adult population using data from NHANES 2003–2014. With this study, the authors demonstrated a positive association with heart failure, CAD, angina pectoris, MI, and CVDs, which was more evident in males [104].

On the other hand, hypertension is a risk factor for CVDs and has also been associated with high levels of BPA. Shankar and Teppala (2012) defined hypertension as “blood pressure-reducing medication use and/or blood pressures >140/90 mm of Hg” [112]. With regard to this, these authors analyzed the relationship between urinary levels of BPA and hypertension in a Multi-ethnic Sample of U.S. Adults. The study was also conducted with participants from NHANES 2003–2004. In agreement with the previous studies mentioned, the results demonstrated a positive association between elevated urinary BPA levels and hypertension, independent of traditional risk factors (OR = 1.50; 95% CI = 1.12–2.00, *p* = 0.007) [112]. Moreover, Bae et al., in 2012, investigated the associations of BPA exposure with HRV and blood pressure (BP) in elderly citizens in Seoul, and demonstrated that urinary BPA was associated negatively with HRV and positively with BP. Furthermore, the authors also found an association with hypertension (OR = 1.27; CI 95% = 0.85–1.88 [113]. Later, in 2015, the same authors also analyzed a possible association between exposure to BPA (from consumption of canned beverages) and BP and HRV. The main findings were that consuming canned beverages (and the consequent increase in BPA exposure) is related to BP increase, but differences in HRV were not found [114]. In the same year, Aekplakorn et al. also determined the association of serum BPA with hypertension in the Thai Population. The authors demonstrated that there is an association between serum BPA levels with hypertension in women (OR = 2.16; 95% CI = 1.31–3.56) [115].

From the studies mentioned above, it is clear that the NHANES dataset has been widely used to draw conclusions about BPA exposure and adverse cardiovascular health outcomes. However, in 2012, La Kind et al. questioned whether this approach is the most appropriate, especially for a chemical with a short physiological half-life such as BPA, and chronic diseases with multifactorial etiologies. With regard to this, analyzing four datasets (2003/04; 2005/06; 2007/08 and 2009/10) and selecting *a priori* consistent methods, the authors tried to clarify the association between urinary BPA concentrations and diabetes, CAD, and/or MI. After adjustment for confounding factors, the results showed no association between urinary BPA levels and heart disease and diabetes, thus, not supporting previous studies wherein different methods and definitions were used. Thus, the authors suggested that using the NHANES data to study the association of BPA with CVDs is not appropriate, and may lead to inconsistent results due to this discrepancy between methodologies. Further epidemiological studies should be conducted to toxicologically evaluate the role of BPA in the association with CVD development [117], as the experimental design of the epidemiological studies developed so far is constraining our clear comprehension of this topic [118]. In addition, Olsén et al. also assessed the associations between circulating levels of BPA and coronary risk in an elderly population [119] using the Framingham risk score to predict CVD risk. Contrary to previous studies, but corroborating the studies of La Kind et al. [117,118], the authors found no strong associations between BPA and FRS in the population, thus suggesting that BPA exposure is not a risk factor for CVDs [119].

In summary, the set of epidemiological studies presented suggests that BPA exposure seems to be related to the development of CVDs or risk factors for CVDs, such as hypertension. Most studies are conducted using NHANES data, evidencing that this causal relationship is consistent with higher urinary BPA levels. The causal relationship with diabetes has also been established. However, due to the discrepancy in methodologies performed (including exclusion criteria, definitions, and scoring systems) caution is needed in their comparative analysis.

#### 5.2.2. Pregnancy Exposure

HDPs are one of the leading causes of maternofetal mortality and morbidity. Exposure to EDCs is suspected to increase BP, but few studies have investigated the impact of these chemicals on pregnant women [120]. Due to BPA being suspected to cross the placenta during pregnancy, possibly affecting children’s health, Bae et al. also analyzed maternal urinary concentrations of BPA during midterm pregnancy (around 20 weeks) and in the BP of children at age 4. The authors demonstrated that there exists a positive association between the diastolic (and not systolic) BP of the children with a maternal urinary concentration of BPA above a threshold level (4.5 μg/g creatinine) [121]. Moreover, Warembourg et al. also found that BPA exposure was associated with a significant decrease in systolic and/or diastolic BP during pregnancy (most frequently observed during the second trimester) [120]. Recently, in 2020, Sol et al. also evaluated the association between maternal BPA urine concentrations during pregnancy and BP in childhood in a population-based prospective cohort study. The authors demonstrated that this association is sex-dependent, as evidenced by higher BP in boys after fetal BPA exposure. However, these results highlight the need for further studies to unravel the underlying mechanisms and long-term outcomes [122].

According to the recently reviewed studies, BPAs (as well as phthalates) are the class of EDCs that appear to mostly harm the fetoplacental vasculature during pregnancy and have been associated with the development of pregnancy complications such as preeclampsia (PE) [51]. However, these specific exposure–response relationships are not yet fully established [123]. In 2014, Leclerc et al. were the first authors to demonstrate that there was an association between PE and BPA accumulation in the placenta. The authors found that placentas from women with PE had a higher accumulation of this EDC compared to placentas from normotensive women [124]. In the following year, Fergunson et al. also observed that there is an association between urinary BPA concentrations and angiogenic biomarkers during pregnancy, which may be indicative of this pathology through placental disruption development and/or function during gestation [125]. Concordantly, Spagnoletti et al. also demonstrated that BPA acts on trophoblasts and alters physiological processes involved in normal placental development [97], and Cantonwine et al. also reported that early pregnancy (~10 weeks gestation) appears to be a window of increased susceptibility for the development of this HDP associated with BPA exposure [126]. Furthermore, in 2017, Philips et al., in their findings, suggested that there is probably a greater association of bisphenols in early pregnancy with a risk of PE than with gestational hypertension (as no significant changes in BP were found) [127]. The same authors also recently described that early exposure to BPA during pregnancy appears to be related to changes in cardiometabolic health later in life [128].

Additionally, the postpartum period may be a vulnerable life stage for a woman’s cardiometabolic health [129]. According to the recent review by Lorigo and Cairrao, considering the postpartum period is equally as important as considering the pregnancy period when talking about endocrine disruption. The associations between elevated levels of EDCs in general, including BPA, and vascular changes in children or adults are not yet demonstrated; they require further study, because pregnancy and postpartum are both susceptible windows of endocrine exposure. Thus, further studies must be developed to clarify these mechanisms and, thus, understand whether the risk of CVDs is temporary or permanent in later life [51]. With regard to this, in 2020, Perng et al. analyzed the effect of BPA during pregnancy on weight from delivery through 1 year postpartum among 199 women in Mexico City. Through their prospective cohort study, the authors demonstrated that prenatal exposure to BPA is inversely associated with weight at delivery, but there exists a slower rate of weight loss through the first postpartum year [129]. However, to our knowledge, these authors were the only ones to evaluate the effects of BPA in the postpartum period.

Therefore, taken together, these studies indicate that there is an environmental contribution by EDCs in the development of HDPs; therefore, urgent action should be taken to decrease exposure to these emerging compounds to reduce the mortality and morbidity associated with these conditions.

**Table 5:** Summaries of the disruptive effects of BPA in human epidemiological studies ^1^.

Topic	Studied Population	Gender	Concentration	Observed Effects	References
Cardiovascular diseases	NHANES from 2003–2004 1455 adults aged 18 through 74 years	694 men 761 women	4.53 ng/mL (urinary) 4.66 ng/mL (urinary)	▪ Association between CVDs and elevated urinary BPA levels ▪ Association between higher BPA concentrations and diabetes	[103]
	NHANES 2003–2006 data, separately between 2003/2004 and 2005/2006 and pooled n = 1455 (2003/04) and n = 1493 (2005/06) adults aged 18–74 years	694 men and 761 women in 2003/04 720 men and 773 women in 2005/06	2.49 ng/mL (urinary) 1.79 ng/mL (urinary)	▪ Urinary BPA concentrations were smaller for the 2005/2006 data than those from 2003/3004 ▪ Association between higher BPA levels (2003/3004 data) and CAD, but not with diabetes. ▪ Data pooled showed an association between higher BPA concentrations and CAD and diabetes	[108]
	Population-based Prospective Investigation of the Vasculature in Uppsala Seniors study (1016 subjects all aged 70)	510 women and 506 men	3.76 ng/mL (serum)	▪ Elevated levels of BPA were related to the echogenicity of the plaques, suggesting a role for BPA in atherosclerosis	[105]
	758 incident CAD cases and 861 controls followed for 10.8 years from the European Prospective Investigation of Cancer—Norfolk, UK	534 men and 327 women in control group 501 men and 257 women in CAD group	Control vs. CAD group (1.24 ng/mL vs. 1.35 ng/mL) (urinary)	▪ Urinary BPA concentrations were low ▪ Increased BPA concentration was associated with incident CAD	[106]
	591 patients participating in The Metabonomics and Genomics in Coronary Artery Disease study in Cambridgeshire, UK	120 controls (62 women and 58 men) 385 patients (301 men and 84 women) 86 Intermediate diseases (43 men and 43 women)	Control vs. CAD vs. intermediate (2.13 vs. 3.82 vs. 3.31 ng/mL) (urinary)	▪ Association between BPA exposure with grades of severity of CAD on angiography ▪ Higher urinary concentration of BPA in patients with severe CAD compared to patients with normal coronary arteries	[107]
	745 participants in the NHANES 2003–2004	361 women and 392 men	2.30 ng/mL (urinary)	▪ A positive association between increased levels of BPA with DBP	[110]
	88 DCM patients and 88 age-and gender-matched healthy controls	59 men and 29 women with DCM 55 men and 33 women Control	DCM vs. control group (6.9 ± 2.7 vs. 3.8 ± 1.9 ng/mL) (serum)	▪ Higher levels of BPA in DCM patients compared with the healthy group	[109]
	NHANES 2003–2014 (n = 9139, aged ≥20 years)	4467 men and 4672 women	-	▪ Positive association with heart failure, CAD, angina pectoris, MI, and CVDs, which was more evident in males	[104]
Hypertension	1380 subjects from NHANES 2003–2004	700 women and 680 men (580 with hypertension)	1.5–4.0 ng/mL (urinary)	▪ A positive association between elevated urinary BPA levels and hypertension	[112]
	560 noninstitutionalized elderly citizens from August 2008 to August 2010 in Seoul from Korean Elderly Environmental Panel Study	521 participants were included (138 men and 383 women)	(men) and 1.3 (women) μg/g of creatinine (urinary)	▪ Urinary BPA was associated negatively with HRV and positively with BP ▪ BPA was associated with hypertension	[113]
	60 noninstitutionalized elderly participants, who were aged ≥60 years between February 2014 and March 2014	60 participants, 56 were women and 4 male	1.13 ± 1.76 μg/L (urinary)	▪ Association between exposure to BPA with BP increased, but differences in HRV were not found	[114]
	A subsample of 2558 randomly selected from the Thai National Health Examination Survey IV, 2009	1275 men and 1283 women	0.35 ng/mL (men) and 0.33 ng/mL (women) (serum)	▪ Association between serum BPA levels with hypertension in women	[115]
Pregnancy exposure	645 children at the age of 4 who were born from women who participated, midterm during their pregnancy, in a birth cohort study from August 2008 to July 2011	486 mother–child pairs were included in the present analysis	Maternal urinary: 0.9 μg/L (urinary)	▪ A positive association between diastolic (and not systolic) BP of the children with the maternal urinary concentration of BPA	[121]
	152 female volunteer participants in the Human Early-Life Exposome project	152 pregnant women	3.1 μg/g creatinine (urinary)	▪ BPA exposure was associated with a significant decrease in systolic and/or diastolic BP	[120]
	1064 mother-child pairs/childhood at a mean age of 9.7 years old	1064 mother-child pais	6.0 nmol/L (boys) and 7.2 nmol/L (girls) (urinary)	▪ Higher BP in boys after fetal BPA exposure	[122]
	58 pregnancies, including 35 normotensive and 23 preeclamptic women	35 normotensive pregnant women and 23 preeclamptic pregnant women	Control vs. PE (3.00 vs. 2.80 ng/mL–maternal serum; 2.17 vs. 2.23 ng/mL–fetal serum; 3.00 vs. 9.40–placental homogenate)	▪ Placentas from women with PE had a higher accumulation of this EDC compared to placentas from normotensive women	[124]
	A nested case-control population consisting of 130 mothers who delivered preterm and 352 who delivered term from a prospective birth cohort	130 women and 352 controls	7.08% change (adjusted with soluble fms-like tyrosine kinase-1) (urinary)	▪ Association between urinary BPA concentrations and angiogenic biomarkers during pregnancy	[125]
	A nested case-control study of preterm birth was performed in 2011 from women enrolled in a prospective birth cohort study at Women’s Hospitals in Brigham and in Boston (included 50 cases of PE)	482 women (50 with PE)	Cases vs. control (1.56 vs. 1.38 ng/mL) (urinary)	▪ Early pregnancy (~10 weeks gestation) was a window of increased susceptibility for the development of this HDP associated with BPA exposure	[126]
	1233 women excluding those without any BP measurement or with pre-existing hypertension	1233 women	1.65 ng/mL (urinary)	▪ Association of bisphenols in early pregnancy with a risk of PE	[127]
	1 year postpartum among 199 women in Mexico City	199 women	1.18 ng/mL (urinary)	▪ Prenatal exposure to BPA was inversely associated with weight at delivery, but there exists a slower rate of weight loss through the first postpartum year	[129]

^1^ Legend: BP—blood pressure; BPA—bisphenol A; CAD—coronary artery disease; CVDs—cardiovascular diseases; DCM—dilated cardiomyopathy; EDC—endocrine-disrupting compound; HRV—heart rate variability; MI—myocardial infarction; NHANES—National Health and Nutrition Examination Survey; PE—preeclampsia.

## 6. Conclusions

**Figure 3:**
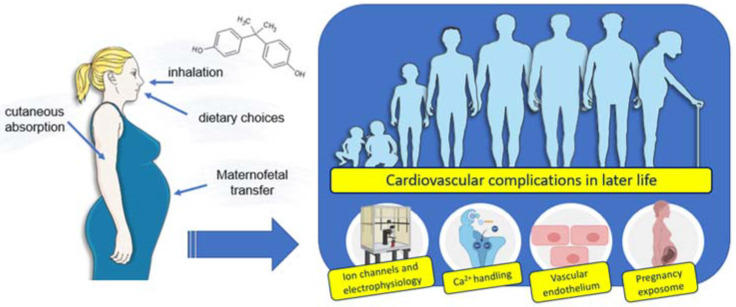
Summary representation of Bisphenol A (BPA) exposure pathways and main mechanisms involved in BPA-induced cardiotoxicity.

The analysis of several studies of BPA-induced cardiotoxicity led us to conclude that exposure to this EDC is associated with an increased prevalence of CVDs. It has become clear that BPA, even acting at lower doses as an EDC, can cause adverse effects on cardiovascular health, especially at more susceptible stages of development, such as pregnancy. BPA exposure impairs not only cardiac electrophysiology, but also Ca^2+^ signaling, leading to arrhythmias and changes in contractile functions (contraction and relaxation) (Figure 3). However, the concentrations used in most in vitro studies are relatively high. Therefore, the choice of concentrations to be used in the in vitro, ex-vivo, and in vivo studies is one of the greatest challenges of toxicology, and this choice should always be dependent on the concentrations detected in epidemiological studies (a concentration range from 1.6 to 946 μg/L) [51] and environmental studies (effluent ranging from non-detect to 370 μg/L, soil ranging from <0.01 to 1000 μg/kg, and sediments ranging from 3400 to 20,136 μg/kg DW [130]. The use of higher concentrations (20–200 times higher than therapeutic C_max_) in in vitro studies is consensual. These concentrations are also designated using pharmacological concentrations and cause more accurate results than lower concentrations [131]. The ranges of these pharmacological concentrations are those that are required to cause effects at the cellular level, and are normally bigger than those required to cause harmful effects in vivo. This interrelationship is called the in vitro–in vivo scaling factor. However, this factor depends mainly on the mechanism of action of the substance, the metabolization route, and the mechanism of toxicity. Thus, only these high concentrations may be predictive of the effects induced by these substances [131,132]. In summary, the use of a high concentration range is crucial to determine the occurrence of toxic effects of substances, such as for BPA.

Another very important parameter that remains under great controversy is the daily amount considered safe for human exposure, which is also referred to as the acceptable daily intake (ADI) by the Food and Drug Administration (FDA), and as the reference dose by the Environmental Protection Agency (EPA). This dose is currently at 50 µg/kg/day [133] and assumes that even if a daily exposure of this amount occurs, no adverse effects occur. However, recent studies have shown that the lowest observed adverse effect level (LOAEL), which serves as the reference ADI, is much lower than currently accepted (20,000-fold lower). These studies have led several members of the Endocrine Society to notify the FDA and EPA that there are several incorrect assumptions regarding the calculation of AID for BPA: (1) the responses to BPA are likely to be monotonic, which, in reality, is not the case for EDC; (2) the threshold at which there are no effects, which is also very controversial and questioned, as it is known that it can develop changes in the next generations; (3) a similar response for both sexes, which is also not true for many responses to BPA; and (4) only toxicological guideline studies are valid [133].

Thus, it seems clear that the human current daily exposure is not safe and that the problems arising from the massive use of this EDC continue to be a reality. The controversial studies can be justified through the different experimental approaches that are applied, as well as changes in the levels of doses, duration of exposure, or even the experimental model used. With regard to this, more studies are needed to further clarify the mechanism of BPA toxicity in the cardiovascular system. We emphasize that it is essential to use a wide range of concentrations to adequately address the dose–response relationship of BPA.

Furthermore, as humans are widely exposed to multiple bisphenols, this underscores the need for and importance of studying co-exposure patterns and/or the ‘exposome’, always considering multiple exposures and, thus, the best way to address the adverse effects of this EDC on cardiovascular health. The development of new BPA analogues, as well as the study of the safety of some analogues currently already developed, are a key point in the evolution of research on these compounds. Environmental and human exposure to these compounds should be free of health risks, especially in the most vulnerable groups such as children and pregnant women. Finally, it is also important that public health approaches are taken to clarify which are the best practices for reducing exposure to BPA (e.g., recommending the use of glass containers for food storage, reducing the use of canned products and plastic containers, etc.), thus improving human lifestyles and, consequently, decreasing the risk of BPA-induced cardiotoxicity.

## Figures and Tables

**Table 1:** Summaries of the disruptive effects of BPA in the animal in vitro studies ^1^.

Drugs	Concentration	Animals/Organs/Cells	Results	References
BPA and Penitrem	10 µmol/L 1 µmol/L	Canine coronary smooth muscle cells AD 293 cells	▪ Activated an external current in smooth muscle cells previously inhibited by penitrem ▪ Increased Maxi-K activity	[35]
BPA and/or 17β-estradiol(E2)-	1 nmol/L	Ventricular myocytes and Sprague Dawley adult mice heart and ERβ knockout mice (Erβ^−/−^)	▪ Rapid induced arrhythmogenic effect in females ▪ Pronounced when combined with estradiol ▪ Ventricular arrhythmias ▪ Rapidly altered myocyte Ca^2+^ handling ▪ Increased sarcoplasmic reticulum leak ▪ Ryanodine inhibition of SR Ca^2+^ leak suppressed estrogen-induced triggered activities.	[45]
BPA and/or E2	0.001–1 nmol/L	Rat Sprague Dawley myocytes and female knockout Erβ mice.	▪ Concentration–response curve for stimulatory effects (contractility and arrhythmogenic) of BPA and E2 in female myocytes was inverted-U-shaped ▪ Rapid arrhythmogenic effects	[46]
BPA	1–100 µmol/L	HEK293 cells transfected with Human Cardiac Sodium Channel	▪ BPA induced a dose-dependent tonic block of the human Nav1.5 sodium channel	[43]
BPA or BPA and E2	1 nmol/L	Adult Sprague Dawley rats’ hearts	▪ Increase in the duration of sustained ventricular arrhythmias ▪ Increased ventricular fibrillation duration ▪ Pro-arrhythmic effects of estrogens abolished by MPP combined with PHTPP ▪ Reduced infarction size	[47]
BPA	1 nmol/L	Female rat ventricular myocytes	▪ BPA rapidly activated two parallel signaling pathways, the cAMP/PKA pathway, and the PLC/IP3/Ca^2+^/CAMKII pathway.	[48]
BPA	1–100 μmol/L	Mouse cardiac myocytes	▪ BPA interacted with calcium channels by binding to an external site outside the pore-forming region	[38]
BPA membrane-impermeant BPA-monosulfate (BPA-MS)	100 µmol/L	AD 293 cells expressing α or α + β1 subunits	▪ Increased BK channel activity	[36]
BPA	1–100 μmol/L	HEK 293 cells transfected with CaV3.1-CaV3.3	▪ BPA inhibited T-type calcium channels ▪ Low (nanomolar) concentrations inhibited only a minor part of channels ▪ Micromolar concentrations blocked the channel in both open and inactivated states.	[39]
BPA	0.1 nmol/L^−1^–1 μmol/L	Female rat ventricular myocytes	▪ Inverted-U-shaped dose–response	[40]
BPA	0.001–100 µmol/L	Neonatal rat cardiomyocytes	▪ Reduced Ca^2+^ transient amplitude ▪ Prolonged Ca^2+^ transient release time	[54]
BPA	0.001–100 µmol/L	A7R5 cells from rat aorta	▪ Inhibition of L-type calcium channels	[37]
BPA	100 µmol/L	Neonatal rat cardiomyocytes	▪ Reduced the spontaneous beating rate and increased beat rate variability. ▪ Diminished calcium transient amplitudes, prolonged calcium transient upstroke and duration time.	[50]
BPA	1–100 µmol/L	Zebrafish larvae Zebrafish cell lines	▪ BPA, BPAF, and BPC were agonists with different potencies for the three zebrafish estrogen receptors	[49]
BPA and/or PFOS	25 μmol/L for 14 days	Rat cardiomyocytes	▪ Increased level of total collagen and dynamin-associated protein 1 mRNA ▪ Decrease in mitochondrial length and ATP level	[52]
BPA	0–10 µmol/L BPA for 24 h	Murine aortic ECs (MAECs) and H9c2 cells.	▪ Increased the expression of RIP 3 ▪ Increased expression of inflammatory cytokines	[53]
BPA	1–100 μmol/L	hiPSC-CM	▪ BPA exposure inhibited Ca^2+^ transients and cardiac contraction ▪ BPA exposure affected Cav1.2, Nav1.5, and hERG channel activity.	[41]
BPA Bisphenol S Bisphenol F	0.0–100 µmol/L	hiPSC-CM	▪ BPA was the most potent inhibitor of the sodium channel, L-type Ca^2+^ channel, and hERG channel current	[42]

^1^ Legend: BPA—bisphenol A; Ca^2+^—calcium; hiPSC-CMs—human-induced pluripotent stem-cell-derived cardiomyocytes; PFOS—perfluorooctane sulfonate.

**Table 2:** Summaries of the disruptive effects of BPA in animal ex vivo studies ^1^.

Drugs	Concentration	Animals/Organs/Cells	Results	References
BPA	0.1–100 μmol/L	Adult albino rats of Charles Foster strain	▪ Depressed the contractility of spontaneously beating atria ▪ Decreased the rate and force of atrial contractions simultaneously.	[55]
BPA	0.1–100 µmol/L	Sprague Dawley rat adult hearts	▪ Prolonged PR segments ▪ Decreased epicardial conduction velocity ▪ Prolonged action potential duration ▪ Delayed atrioventricular conduction ▪ Prolonged QRS intervals ▪ Dropped ventricular beats	[30]
BPA	0.001–100 µmol/L	Sprague Dawley rat hearts	▪ Decreased left ventricular developed pressure and inotropy in a dose-dependent manner ▪ Reduced contractile performance ▪ Altered Ca^2+^ handling in the heart and neonatal cardiomyocytes	[54]
BPA	0.001–100 µmol/L	Male Wistar aorta rats	▪ Rapid and concentration-dependent relaxation of rat aorta	[37]
BPA	10 µmol/L and 25 µmol/L	Goldfish (C. auratus) adults hearts	▪ Impaired Frank–Starling response ▪ Structural myocardium changes ▪ Increased cardio-somatic indices ▪ Altered oxidative state ▪ Negative chronotropic effect	[56]

^1^ Legend: BPA—bisphenol A; Ca^2+^—calcium.

**Table 3:** Summaries of the disruptive effects of BPA in the animal in vivo studies ^1^.

Drugs	Concentration	Animals/Organs/Cells	Results	References
BPA	0.5, 5.0 and 200 µg/kg day	Rats	▪ Altered cardiac structure/function and blood pressure ▪ Increased body weight, BMI, and body surface area ▪ ERCA2a, NCX1, and CASQ2 expression was altered sex-specifically	[57]
BPA	50 μg/kg body weight/day–12 weeks	ApoE^−/−^ male mice	▪ Increase in non-HDL cholesterol ▪ Increased HDL cholesterol ▪ Increased the expression of TNF-α and IL-6	[59]
BPA	4 nmol/L–400 µmol/L	Mice CD1	▪ BPA induced high blood pressure and impaired carotid relaxation in mice ▪ BPA regulated blood pressure by inducing AngII/CaMKII-α uncoupling of eNOS	[58]
BPA or EE	0.15–5000 µg/kg/day	CD1 mice	▪ Decreased systolic blood pressure ▪ Dimorphic sexual changes in extracellular matrix composition ▪ Altered autonomic tone	[60]
BPA	25 mg/kg 10 mg/kg	Adult male Wistar albino rats	▪ Increase in malondialdehyde ▪ Decrease in catalase activity ▪ Significant decrease in reduced glutathione and acetylcholinesterase activity. ▪ Decrease in nitric oxide level ▪ Increase in body weight	[61]
BPA	25 ng/mL–5 µg BPA/kg BW/day	C57bl/6n mice	▪ Collagen and αSMA expression were reduced by 50% ▪ Reduced cardiac remodeling after an experimental myocardial infarction	[62]
BPA	100 and 2000 µg/L	Zebra fish	▪ Increased rate of heart failures of progeny up to F2 ▪ Decreased gene expression of cardiac development in F1 embryos ▪ cardiac edema, incorrect looping, and showed disorganized heart walls in F1 and F2	[74]
BPA and/or hipóxia	0.25, 1 and 5 mg/L 1.0 mg O_2_/L	Zebra fish embryos	▪ Induced severe bradycardia ▪ Reduced cardiac output	[73]
BPA or EE	BPA (2.5–25,000 µg/kg day) EE (0.05 or 0.5 µg/kg/day)	PND21, PND90, PND180 Sprague Dawley rat	▪ Heart weight gain ▪ Increased fibrosis ▪ Increased incidence and severity of progressive cardiomyopathy ▪ Myocardial degeneration was observed in both males and females at PND21 and PND90	[63]
BPA	5, 50, and 500 μg BPA/kg bodyweight/day	Juvenile female Fischer 344 rats	▪ Increased mRNA expression of Vegf, Vegfr2, eNos, and Ace1 in rat heart	[65]
BPA	50 mg/kg	Adult PXR-Humanized Mice	▪ hPXR-mediated epigenetic regulation of aortic fatty acid transporter CD36 expression in the aorta ▪ Increased atherosclerosis	[66]
BPA	0.1 and 1.0 mg/L	Zebrafish embryos	▪ Induced GFP fluorescence expression in heart valves ▪ ERE activation via estrogen receptor 1	[76]
BPA and/or hypoxia	0.001–100 µg/L	Zebrafish larvae	▪ Decreased red blood cell velocity and outer diameter of the caudal vein	[75]
BPA	0.5, 5, 50 µg BPA/kg body weight	BALB/c Mice	▪ Increased viral myocarditis and pericarditis ▪ Increased CD4+ T cells, IFNγ, IL-17A, TLR4, caspase-1, and IL-1β in the heart	[67]
BPA and/or EGCG	2000 and 4000 µg/L BPA 50 and 100 µmol/L EGCG	Zebrafish embryos	▪ Impaired cardiogenesis ▪ Altered gene expression of cardiomyocyte differentiation and histone acetylation	[78]
BPA or metabolite MBP	100 and 1000 µg/L 2.5 and 25 µg/L	Embryo—larval zebrafish	▪ Ultrastructural changes in atrioventricular valve sections ▪ Altered gene expression responsible for the development and function of the cardiac valve. ▪ Narrowing and lack of collagen in the extracellular matrix	[77]
BPA	1–100 µmol/L	Zebrafish larvae	▪ Activation of GFP expression in heart (zfERα-dependent) at lower concentrations.	[49]
BPA	Orally exposed to 4 × 10^−5^ mol/L of BPA in drinking water for 4, 8, and 16 weeks	Wild-type CD1 mice	▪ Increased heart rate ▪ Prolonged PQ interval and PR segment ▪ Cardiac contractility impaired ▪ Decreased ejection fraction ▪ Diastolic and systolic interventricular septum thickness (IVSd) were increased ▪ Increased systolic and diastolic blood pressure	[53]
BPA	2 and 100 μg/L BPA	Pregnant rats	▪ Increased septal thickness in the ventricular tissue ▪ Increased myocardial collagen content	[52]
BPA EGCG	100 and 2000 μg/L BPA 50 µmol/L EGCG	Zebrafish male	▪ Induced an increase in sperm histone acetylation ▪ Modified the chromatin structure of crucial genes for heart development	[79]
BPA	BPA (0.25–12 mg L^−1^)	Zebrafish embryos	▪ Stopped intersegmental vessel (ISV) growth ▪ Delayed common cardinal vein (CCV) remodeling ▪ Decreased subintestinal vessels (SIVs)	[80]
BPA	10 µmol/L and 25 µmol/L	Goldfish (C. auratus) adult hearts	▪ Impaired Frank–Starling response. ▪ Structural myocardium changes ▪ Increased cardio-somatic indices ▪ Altered oxidative state	[56]
BPA and metabolite MBP	7 d exposure to 10 μg/L of BPA and MBP	Male Cyprinodon variegatus fish	▪ Induced proteome alterations typical of estrogenic EDC ▪ Increased acidic protein (WAP)	[81]

^1^ Legend: BPA—bisphenol A; EE—17α- ethinylestradiol; EGCG—epigallocatechin gallate; MBP—4-Methyl-2,4-bis(p-hydroxyphenyl)pent-1-ene.

## Data Availability

Not applicable.

## Abbreviations

Ang-II	angiotensin II
BP	blood pressure
BPA	bisphenol A
BPAF	bisphenol AF
BPC	bisphenol C
Ca^2+^	calcium
CAD	coronary artery disease
CnAβ	calcineurin
CVDs	cardiovascular diseases
DCM	dilated cardiomyopathy
DRP1	dynamin-related protein 1
EC	endothelial cells
EDC	endocrine-disrupting compound;
EDHF	endothelium-derived hyperpolarizing factor
ER	estrogen receptors
ET-1	endothelin 1
GPR30	G-protein-coupled estrogen receptor
HDPs	hypertensive disorders of pregnancy
HEK	human embryonic kidney
hiPSC-CMs	human-induced pluripotent stem-cell-derived cardiomyocytes
HRV	heart rate variability
HUVECs	human umbilical vein endothelial cells
IC50	half-maximal inhibitory concentration
iPSCs	induced pluripotent stem cells
IR	ischemia–reperfusion
K^+^	potassium
MAP	mean atrial pressures
MBP	4-Methyl-2,4-bis(p-hydroxyphenyl)pent-1-ene
MI	myocardial infarction
MOA	mechanisms of action
Na+	sodium
NHANES	National Health and Nutrition Examination Survey
NO	nitric oxide
PAD	peripheral artery disease
PE	preeclampsia
PFOS	perfluorooctane sulfonate;
PGI-2	prostacyclin
PLB	phospholamban
PPAR-γ	peroxisome proliferator-activated receptor gamma
RIP3	Rat Receptor-Interacting Protein 3
SMC	smooth muscle cells
TxA2	thromboxane
U.S.	United States
XME	xenobiotic-metabolizing enzymes
